# L-Glutamine and Survival of Patients with Locally Advanced Head and Neck Cancer Receiving Chemoradiotherapy

**DOI:** 10.3390/nu15194117

**Published:** 2023-09-23

**Authors:** Takae Tsujimoto, Masafumi Wasa, Hidenori Inohara, Toshinori Ito

**Affiliations:** 1 Department of Clinical Pharmacy, Faculty of Pharmaceutical Sciences, Kobe Gakuin University, 1-1-3 Minatojima, Chuo-ku, Kobe 650-8586, Japan; 2 Department of Pediatric Surgery, Osaka University Graduate School of Medicine, 2-15 Yamadaoka, Osaka 565-0871, Japan; 3 Department of Otorhinolaryngology-Head and Neck Surgery, Osaka University Graduate School of Medicine, 2-15 Yamadaoka, Osaka 565-0871, Japan; 4 Osaka Center for Cancer & Cardiovascular Disease Prevention, 1-6-107 Morinomiya, Johtou-ku, Osaka 536-0025, Japan

**Keywords:** L-glutamine, head and neck cancer, chemoradiotherapy, overall survival, progression-free survival, glutathione, overall response rate, oral glutamine supplementation, mucositis, reactive oxygen species

## Abstract

We previously reported that L-glutamine reduces the severity of mucositis caused by chemoradiotherapy in patients with head and neck cancer. However, the impact of glutamine on the anti-tumor effect of chemoradiotherapy remains controversial. This study, which included 40 patients, investigated whether L-glutamine influences survival. Radiation therapy (total: 66 or 70 Gy), cisplatin, and docetaxel were co-administered for a period of 6 weeks. Patients were randomly assigned to receive either glutamine (glutamine group, *n* = 20) or placebo (placebo group, *n* = 20) during the entire course of chemoradiotherapy. We compared the overall survival and progression-free survival rates between the two groups. At 5-year follow-up, 16 (80%) and 13 (72%) patients in the glutamine and placebo groups, respectively, survived (with no significant difference in overall survival [glutamine group: 55.2 ± 12.7 months vs. placebo group: 48.3 ± 21.3 months]). A total of 14 (70%) and 12 (67%) patients in the glutamine and placebo groups, respectively, did not experience disease progression (with no significant difference in progression-free survival [glutamine group: 46.7 ± 19.5 months vs. placebo group: 43.6 ± 25.2 months]). These findings indicate that L-glutamine does not influence the survival of patients with locally advanced head and neck cancer receiving chemoradiotherapy.

## 1. Introduction

The free amino acid L-glutamine exhibits the greatest abundance among amino acids in humans. Specifically, it accounts for approximately 60% of the total amount of amino acids. This amino acid is the main source of energy and essential precursor for nucleotide biosynthesis in numerous types of rapidly proliferating cells, namely intestinal epithelium, fibroblasts, lymphocytes, and macrophages [1,2,3,4]. Glutamine is, therefore, considered a conditionally essential amino acid in these types of cells. It is utilized as a substrate for the synthesis of glutathione and has antioxidative properties [5]. Exposure to high levels of stress markedly impairs the synthesis of glutamine in the human body, thereby leading to reduced levels of glutamine in plasma. These conditions adversely affect the mucosal immunity and decrease the amount of glutamine released from muscle tissue. Wasa et al. stated that glutamine is a key factor in the biosynthesis of DNA and proteins in cell lines of solid tumors [6]. Of note, tumor cells are characterized by rapid proliferation, and this process requires amino acids for DNA and protein biosynthesis, as well as energy production.

The use of cytotoxic therapy in patients with advanced cancer may lead to the development of glutamine deficiency [7]. It has been reported that supplementation with exogenous glutamine can prevent and alleviate mucosal damage [8,9,10]. According to Nose et al., the administration of bolus enteral glutamine in rats may prevent the occurrence of cisplatin-induced damage to the intestinal mucosa by enhancing glutamine transport [11]. The results of our recent study revealed that supplementation with oral glutamine in patients suffering from head and neck cancer reduces the severity of mucositis induced by chemoradiotherapy [12]. Topkan et al. orally administered glutamine to reduce acute radiation-induced esophagitis in patients with stage 3B non-small-cell lung cancer who had received chemoradiotherapy. They found that glutamine significantly reduced the severity of acute radiation-induced esophagitis in the glutamine-treated group compared to the non-glutamine-treated group. However, there are concerns that glutamine may have an effect on tumor growth and, thus, negatively affect the outcomes of cancer therapy [13]. Indeed, glutamine released from skeletal muscle is captured by tumors [14], and human tumors consume glutamine at a markedly higher rate than normal healthy tissues [15,16,17]. These data suggest that supplementation with glutamine enhances tumor growth in patients with cancer. 

Klimberg et al. demonstrated that supplementation with glutamine did not increase tumor DNA content. This finding indicated that supplementation with glutamine may be useful in non-tumor tissues (e.g., muscle), the intestinal epithelium, and cells (e.g., fibroblasts, lymphocytes, and macrophages) [18]. Evidence from several studies showed that glutamine is not associated with enhancement of tumor growth and does not negatively influence the outcomes of various kinds of treatment against cancer [19,20]. As shown by Topkan et al., supplementation with oral glutamine treatment of patients with locally advanced non-small-cell lung cancer receiving chemoradiotherapy did not result in significantly different cancer-related clinical outcomes, overall survival, and progression-free survival, compared to the no-glutamine treatment group [19]. In a previous study, we compared tumor size at 10 weeks following the completion of chemoradiotherapy in patients with head and neck cancer. The results showed that there was no significant difference in the overall response rate, which is the percentage of patients who achieved a complete or partial response between the glutamine and placebo groups [12]. Another study involving patients with breast cancer demonstrated that the reduction in the size of the tumor did not differ significantly between the glutamine and placebo groups [20]. However, the effects of supplementation with glutamine on the treatment of cancer remain to be fully investigated.

Therefore, the goal of the present study was to explore whether glutamine influences cancer therapy in patients with locally advanced head and neck cancer.

## 2. Materials and Methods

### 2.1. Patients

The patient characteristics, chemoradiotherapy protocol, and glutamine administration were described in detail in a previous report [12]. Briefly, between May 2010 and June 2013, 40 patients with head and neck cancer participated in a randomized, placebo-controlled, double-blind trial. The purpose of the study was to investigate whether glutamine reduced the severity of mucositis induced by chemoradiotherapy. Two patients with nasopharyngeal cancer were excluded from the analysis. These 38 patients were randomly classified into a glutamine (*n* = 20) or placebo (*n* = 18) group. All patients have squamous cell carcinoma The study population included 17 men and 3 women in the glutamine group and 16 men and 2 women in placebo group. The mean age of the patients was 60.5 ± 10.8 years in the glutamine group and 64.1 ± 4.9 years in the placebo groups. Matching of the two groups for primary tumor site, tumor stage, body mass index, and performance status was performed according to the scale established by the Eastern Cooperative Group. All patients received radiation therapy with a total of 66 or 70 Gy (rate: 2 Gy/fraction/day). A total of five fractions were delivered each week. The patients were treated with intravenous co-administration of cisplatin (dose: 20 mg/m^2^) and docetaxel (dose: 10 mg/m^2^) once per week for a total of 6 weeks. Throughout the course of chemoradiotherapy, patients in the glutamine and placebo groups received 10 g of glutamine or placebo orally, three times per day. The two groups were compared in terms of the severity of mucositis. Of note, the total doses of radiation therapy, cisplatin, or docetaxel did not differ significantly between the two groups. The study was performed in accordance with the tenets of the Declaration of Helsinki. The protocol of this investigation was approved by the Institution Review Board of Osaka University Hospital (approval number: 09180) approval 16 October 2009. All patients provided written informed consent before they underwent any procedures related to the study. This trial was registered in the University Hospital Medical Information Network Clinical Trials Registry (UMIN000003991).

### 2.2. Survival

The rates of overall survival and progression-free survival were compared between the two groups. Patients were followed up for 5 years. Overall survival was defined as the period of time from the day of randomized assignment to that of death due to any cause. Progression-free survival was defined as the period of time from the day of randomized assignment to that of either local/regional or distant progression or death due to any cause. 

### 2.3. Statistical Analyses

Kaplan–Meier survival curves were produced for overall survival and progression-free survival. Comparison of the survival rates was performed using stratified log-rank tests. The analyses yielded two-tailed *p*-values, and a *p*-value < 0.05 denoted statistical significance. EZR software (Saitama Medical Center, Jichi Medical University; https://www.jichi.ac.jp/saitama-sct/SaitamaHP.files/statmedEN.html (accessed on 22 September 2023); Kanda, 2012, Saitama, Japan), which is a graphical user interface for R (The R Foundation for Statistical Computing, Vienna, Austria; version 2.13.0), was used for all statistical analyses. This software is a modified version of R commander (version 1.6-3), which includes features commonly utilized in biostatistical analyses.

## 3. Results

### 3.1. Overall Survival and Progression-Free Survival 

**Figure 1:**
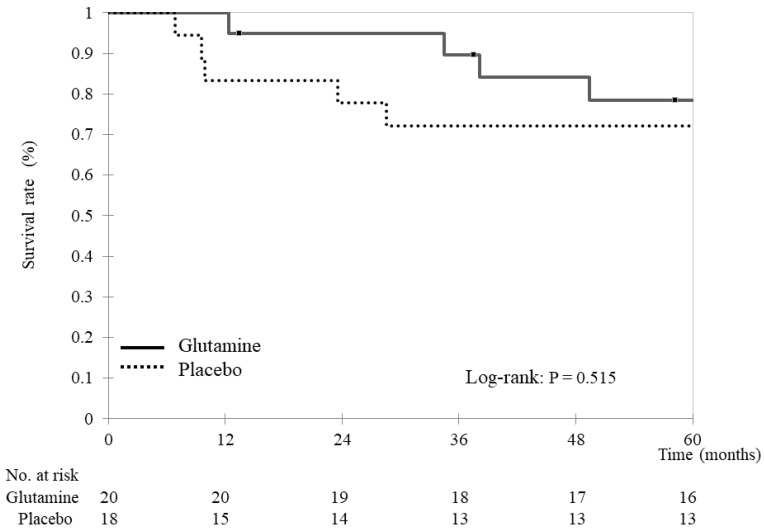
Overall survival Kaplan–Meier survival curves for overall survival. Overall survival was compared between the glutamine and placebo groups using stratified log-rank tests. The analyses yielded two-tailed *p*-values, and *p*-values < 0.05 indicate statistically significant differences.

At 5 years, 29 patients (76%) had survived (16 and 13 in the glutamine and placebo groups, respectively). The two groups exhibited similar results in terms of overall survival (glutamine group: 55.2 ± 12.7 months vs. placebo group: 48.3 ± 21.3 months, *p* = 0.583, Figure 1). In the glutamine group, the causes of death were cancer-related (three patients) and unknown (one patient). In the placebo group, the causes of death were cancer-related (four patients) and suicide (one patient).

**Figure 2:**
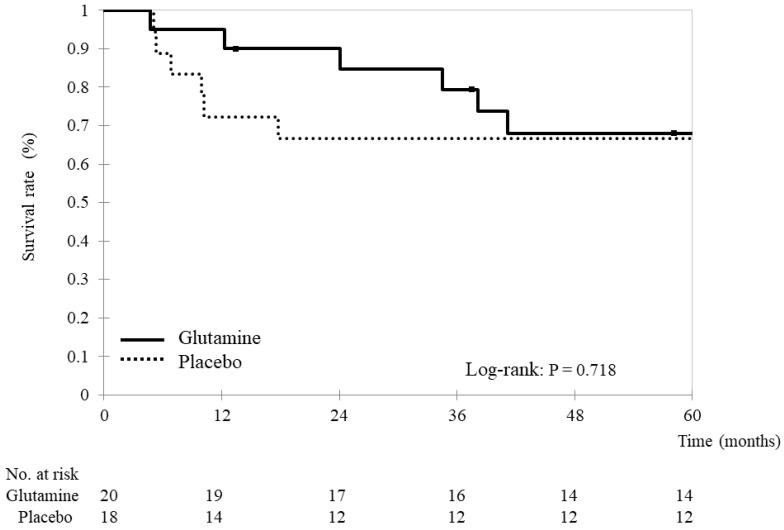
Progression-free survival Kaplan–Meier survival curves for progression-free survival. Progression-free survival was compared between the glutamine and placebo groups using stratified log-rank tests. The analyses yielded two-tailed *p*-values, and *p*-values < 0.05 indicate statistically significant differences.

Of all patients, 26 (68.4%) survived without disease progression (14 and 12 in the glutamine and placebo groups, respectively). The two groups did not demonstrate a significant difference in progression-free survival (glutamine group: 46.7 ± 19.5 months vs. placebo group: 43.6 ± 25.2 months, *p* = 0.682) (Figure 2). 

### 3.2. Interruption and Salvage Surgery 

The proportion of patients who interrupted treatment for more than one day was 45% (9/20) in the glutamine group and 39% (7/18) in the placebo group, with no significant difference between the two groups (*p* = 0.519). Salvage surgery was performed in 10% (2/20) in the glutamine group and 6% (1/18) in the placebo group, with no significant difference between the two groups (*p* = 1.000). Subsequent therapy after chemoradiotherapy was not performed until recurrence or metastasis in both groups. TPF therapy (docetaxel + cisplatin + fluorouracil) was often administered for recurrence or metastasis in both arms.

## 4. Discussion

In this study, glutamine was administered orally to patients with locally advanced head and neck cancer throughout the course of chemoradiotherapy. Thereafter, the overall survival and progression-free survival recorded in the glutamine and placebo groups were compared. Our analysis did not reveal significant differences in these outcomes between the two groups. This finding demonstrates that the administration of glutamine can be safely implemented without a negative measurable effect on tumor progression and patient survival. 

Thus far, only a few clinical studies have investigated the effects of supplementation with glutamine on cancer treatment and outcomes. In one study, Topkan et al. focused on patients with locally advanced non-small-cell lung cancer undergoing chemoradiotherapy. They discovered that the overall survival, progression-free survival, and local/regional progression-free survival did not differ significantly between the glutamine (30 g per day) treatment group and no glutamine treatment group at a median follow-up of 24.2 months [19]. A study on patients with non-small-cell lung cancer undergoing chemoradiotherapy also compared overall survival and disease-free survival in the glutamine (30 g per day) and non-glutamine groups. After 13.1 months of follow-up, there was no significant difference in overall survival and disease-free survival in the two groups, and multivariate analysis revealed that nodal stage and not receiving glutamine were poor prognostic factors contributing significantly to disease-free survival [21]. In another study, glutamine (30 g per day) was orally administered to patients with breast cancer who were receiving treatment with cyclophosphamide, epirubicin, and 5-fluorouracil. The results showed that the rate of reduction in tumor size did not differ significantly between the glutamine group and placebo group [20]. The present study is the first randomized, placebo-controlled trial evaluating the impact of glutamine on tumor control and the survival of patients with locally advanced head and neck cancer receiving chemoradiotherapy. 

Several studies have demonstrated that glutamine protects normal cells from the toxicity of chemoradiotherapy without adversely affecting the effectiveness of cancer therapy. Rubio et al. constructed a cancer model in rats to examine the effects of treatment with methotrexate. The total amount of methotrexate in tumors exhibited a three-fold increase in the glutamine group compared to the control group. The results suggested that glutamine enhances the sensitivity of tumor cells to chemotherapy [22]. Through a cancer model of rats treated with methotrexate, Rouse et al. showed that the levels of glutathione are decreased in tumor cells but increased in the gut and muscle. These findings implied that the administration of glutamine enhances the selectivity of methotrexate by protecting normal tissues [23]. Numerous studies reported that glutamine exerts a protective effect on the mucosal epithelium [8,9,10,19]. In patients with head and neck cancer undergoing chemoradiotherapy with cisplatin and 5-fluorouracil, the incidence and severity of mucositis significantly reduced in the group of patients who received L-alanyl-L-glutamine intravenously compared to the group of patients who received the same dose of saline as placebo [8]. Bolus administration of enteral glutamine prevents intestinal mucosal damage induced by cisplatin in rats, possibly by increasing the intracellular levels of glutathione [11]. It has been shown that the administration of glutamine-enriched enteral nutrition before and after whole-body radiation therapy prevents intestinal mucosal damage and inhibits bacterial translocation in rats [24]. Oral supplementation with glutamine reduces the incidence rate and duration of radiation-induced acute esophagitis in patients with non-small-cell lung cancer receiving radiation therapy [10,21]. Reducing the dose of anticancer drugs or interrupting radiotherapy or chemotherapy to reduce side effects in chemoradiotherapy can prolong the overall treatment period and lead to the re-growth of the tumor [21,25,26]. In patients with non-small-cell lung cancer, it has been reported that significantly fewer patients in the glutamine-treated group had their chemoradiotherapy interrupted or their overall treatment time prolonged than in the non-glutamine-treated group [19]. This can lead to successful cancer treatment by preventing interruption or prolongation of cancer treatment. These results support our finding that glutamine does not adversely affect the efficacy of cancer therapy, and protects normal cells from the toxicity of chemoradiotherapy. 

Glutathione is the main intracellular antioxidant that protects cells from oxidative damage [27]. Notably, glutamine is a precursor of glutathione. Thus, this relationship underlies our concern regarding the negative impact of glutamine on cancer treatment and clinical outcomes. Anticancer drugs and radiotherapy promote the apoptosis of cancer cells through the generation of reactive oxygen species; however, it has been shown that glutathione removes reactive oxygen species [27]. Therefore, it is hypothesized that glutamine may impede cancer therapy, as it inhibits the apoptosis of cancer cells by increasing the intracellular levels of glutathione. However, glutathione is necessary for the protection of normal cells from the side effects of anticancer therapies, such as mucosal epithelial injury [28]. Klimberg et al. showed that, in mammary carcinogenesis, exogenous glutamine administration promotes the production of glutathione in normal tissues, whereas it decreases the levels of glutathione in tumors [29]. Another study demonstrated that glutamine reduces the levels of glutathione in tumors and decreases the glutathione/oxidized glutathione ratio in cells. This ratio of glutathione/oxidized glutathione is an indicator of oxidative stress, or cytotoxicity, occurring in the cell, and higher levels of oxidized glutathione in the cell indicate that more oxidative stress has occurred in the cell. Therefore, a decrease in the ratio indicates that glutamine administration decreased cytotoxicity. This evidence suggests that exogenous glutamine can be effectively and safely utilized in non-tumor tissues, including muscle and mucous membranes [30]. Exogenous glutamine replenishes the levels of glutathione in tissue, which have been previously depleted by chemotherapy. Through this effect, glutamine protects the mucosal epithelium from oxidative stress [31]. These data suggest that glutamine reduces cytotoxicity in normal tissues by increasing the intracellular levels of glutathione without affecting the efficacy of cancer therapy. 

Glutamine has been used in the fields of bone marrow transplantation and intensive care, such as in patients with postoperative sepsis, multiple traumas, and severe burns [32,33,34]. However, the clinical application of glutamine in oncological diseases is currently limited. Research is required to determine whether the administration of glutamine can protect normal cells from the toxic effects of chemotherapy and radiation therapy without adversely affecting cancer treatment or outcomes. The ability of glutamine to reduce the adverse effects associated with cancer treatment may improve the rate of therapy completion and improve the quality of life of patients.

In several clinical studies demonstrating the relationship between glutamine administration and clinical outcomes, including our present results, the dose of glutamine was 30 g per day orally (10 g three times a day at 10 g per dose) [12,19,20]. To the best of our knowledge, a dosage of 30 g per day seems to be an amount that would reduce side effects without affecting cancer treatment, depending on the carcinoma. 

The main limitation of the present study is that it only included patients with head and neck cancer. The sensitivity to glutamine varies depending on the carcinoma and tissue type. Additional research studies are required to determine whether glutamine influences cancer treatment and outcomes and reduces the incidence rate of treatment-related adverse events in various types of cancer. In our study, 30 g per day of glutamine was administered during the course of chemoradiotherapy. Importantly, the dose of glutamine that reduces the occurrence of adverse events without affecting outcomes remains to be determined in other cancers.

## 5. Conclusions

Our analysis suggests that oral glutamine supplementation in head and neck cancer patients treated with chemoradiotherapy does not have a detectable adverse effect on patient survival. However, this finding is limited to a small group of head and neck cancer patients and large prospective clinical trials with larger patient populations are needed to validate this finding.

## Data Availability

The data generated in the present study may be requested from the corresponding author.
